# Eco-dialysis: fashion or necessity

**DOI:** 10.1007/s11255-020-02393-2

**Published:** 2020-02-01

**Authors:** Monika Wieliczko, Jacek Zawierucha, Adrian Covic, Tomasz Prystacki, Wojciech Marcinkowski, Jolanta Małyszko

**Affiliations:** 1 Department of Nephrology, Dialysis and Internal Medicine, Medical University of Warsaw, Warsaw, Poland; 2 Fresenius Medical Care Polska S.A., Poznan, Poland; 3 Grigore T. Popa’ University of Medicine, Iasi, Romania; 4 Fresenius Nephrocare Polska Sp. Z O.O., Poznan, Poland

**Keywords:** Hemodialysis, Protection of environment, Medical waste management, Natural resource management

## Abstract

Hemodialysis (HD) is one of the resource hungry medical interventions. A huge volume of water (about 500 L) and significant amounts of energy (over 7 kW) are used for a hemodialysis session; over a kilogram of waste is produced during this procedure. Thus, HD contributes to global warming while saving patients’ lives. In this paper, we showed these crucial points in HD treatment and possible ways (e.g. modifications in dialysate flow rate) to reduce environmental impact maintaining therapy standards.

## Introduction

Hundreds of reports and research published annually confirm that the last few decades have seen major changes in the climate prevailing on Earth. A number of studies point to the major role played by humans in these changes. A study by John Cook, which analyzed 11,944 papers on global warming and climate change, published in peer-reviewed scientific journals reported that only 0.7% of the analyzed abstracts questioned the human role in this process, and only 0.3% of the publications considered this role to be uncertain [1]. The vast majority of climatologists, regardless of their assessment on the genesis of changes taking place, do not question the existence of global warming and the role of greenhouse gas emissions (including carbon dioxide), deforestation and other processes in driving global climate change. The observed changes, resulting from the increase in temperature on the Earth, concern the majority of physical and biological systems. An undeniable fact is the shrinking of the cryosphere—the withdrawal of mountain glaciers or the reduction of the sea ice range in the Arctic. Water supply problems for inhabitants of many countries are growing, and areas threatened with drought are constantly increasing. Higher temperature on Earth exerts influence on phenology, shifting the range of plants and animals (towards poles and on higher heights) and intensification of extreme weather phenomena. Climate changes particularly affect the aquatic environment—higher concentrations of carbon dioxide in the atmosphere change the pH of water in the oceans, and the warming of freshwaters has a direct impact on its quality [2]. The changes described above, together with the anticipated population growth, already result in a reduction of resources (water, fossil fuels, food), and in the future may lead to their deficit or total depletion, directly affecting the health and quality of life of the human population. In the human history, the massive climate changes were observed a couple of times. Medieval warming and little ice age are well described by scientists. There are a lot of hypotheses concerning the reasons of these anomalies—some of them indicate the sun activity change, abnormal volcanic activity, and the influence of human activity—deforestation of the land in North America and epidemic diseases [3, 4]. Both Medieval warning and little ice age affected huge areas but cannot be treated as global changes. Currently observed climate changes are global—the warming is observed worldwide [5].

Dialysis is one of the most “resource-intensive” fields of medicine. Over 500 L of water, 7 kW of energy and more than a kilogram of medical waste are consumed during hemodialysis. The aim of this paper is to analyze the impact of dialysis on the environment and to review possibilities to reduce the use of resources necessary for the implementation of treatments.

## Dialysis in the world

In 2010, 2,618,000 patients were treated worldwide, but the actual number of patients requiring this type of therapy ranged from 4,900,000 according to a conservative model to 9,700,000 patients in the high-estimated model. Lack of access to treatment in less developed countries (some countries in Asia and Africa) means that over 2,000,000 people do not receive any treatment. It is also assumed that the number of patients undergoing dialysis will double by 2030 around 5,000,000 patients [6]. At the same time, it is assumed that the number of patients with end-stage kidney disease increases by about 6% per year. Nearly 90% of patients treated with renal replacement therapy undergo extracorporeal blood purification (hemodialysis, hemodiafiltration or their variants) and only about 10–11% have peritoneal dialysis [7]. This percentage is different in different countries—in the United States, the percentage of new patients included in the peritoneal dialysis treatment is about 9–10%, and in Poland or Romania below 5% [8, 9].

The above data indicate that around 600 million hemodialysis procedures are performed annually around the world. Such a large and growing number of treatments year after year make hemodialysis a serious burden on the natural environment. The most important goal of manufacturers of equipment and medical devices should be the development of technologies that allow for the most economical use of resources. On the other hand, providers should be aware of the impact of treatment on the environment and choose solutions that minimize the negative impact on the environment [10].

## Resources used in hemodialysis

### Water

Hemodialysis is a renal replacement therapy technique based on the use of diffusion (or convection and diffusion in the case of hemodiafiltration). The passage of particles from the blood into the dialysate requires a constant flow of appropriately selected dialysis fluid. The average water consumption for one HD treatment can be even about 500 dm^3^. This means that during 1-year treatment for one patient, the hemodialysis therapy requires about 78 m^3^ of water. 270,000,000 m^3^ of water is thus necessary for the treatment of all dialysis patients, which is equal to the annual resource for many poor water countries. Some of the water is irretrievably lost in the technological processes of water treatment. Production of 1 L of ultrapure water for dialysis requires 1.5–1.7 raw water. It means that about 60–70% water (depending on water treatment system) is rejected. What is important—the rejected water is still potable and can be used for different, regular purposes (e.g. for windows and floor cleaning, car washing, dish washing, garden watering). Salvage of the rejected water can be directly translated into cost savings. Every year at least 100,000,000 m^3^ can be saved if we re-use the water rejected by water treatment units only.

The next important and easy to address items are control of the flow of the dialysate and of the use water during priming and rinsing.

### Effect of reduced dialysis fluid flow in hemodialysis on water preservation

Hemodialysis (HD)—a standard of care for patients with end-stage renal disease—is a treatment that removes uremic toxins and excess water from the body and regulates the concentration of electrolytes in plasma. In conventional hemodialysis, the blood is brought into contact with dialysis fluid through membranes in a dialyser. The delivered dose of dialysis is an important predictor of patient outcome [11, 12]. The National Kidney Foundation’s hemodialysis guidelines (DOQI) recommended a target single pool Kt/V (spKt/V) of 1.4 per hemodialysis session for patients treated thrice weekly, with a minimum Kt/V of 1.2, in the absence of residual renal function [13]. Dialysis clearance depends on blood and dialysate flow rates, ultrafiltration flow, dialyser membrane surface, mass transfer coefficient for the solute substances and dialysis time [11]. Since the 1960s, the dialysate flow rate (Qd) has routinely been maintained at 500 mL/min. Thus, HD needs huge amounts of water for dialysate production. In the previous publications, Agar et al. had reported that a 4-h HD with Qd 500 mL/min has a water consumption of about 408 L per individual session treatment, with additional 34 L and 51 L being consumed for a 20-min priming phase and 30-min rinsing phase, respectively. A total water consumption for a single hemodialysis session is nearly 500 L. For worldwide HD population, daily water consumption is around 500 million L [14, 15]. Hemodiafiltration (HDF) additionally uses the phenomenon of convective transport. The original forms of HDF—classical and on-line HDF—need external infusion of controlled amounts of substitution fluid. Classical HDF uses external substitution fluid provided as a sterile solution in plastic bags; HDF on-line (more efficient mode of HDF) prepares the substitution fluid that must be sterile and non-pyrogenic, continuously during the treatment. HDF improves the removal of middle-to-large uremic toxins and since 2012, several randomized trials have compared hemodiafiltration to either low-flux and high-flux HD [16–19]. One of these trials (ESHOL Study) has shown significantly reduced all-cause and cardiovascular mortality with hemodiafiltration online compared to high-flux HD [18]. Now many countries in Europe use these treatment methods for their patients (70% and 50% of HD sessions in Germany and France, respectively). Based on recent controlled trials, high volume substitution is preferred—the minimum threshold convective dose to ensure patient benefits starts at 23 L/1.73 m^2^ per session in post-dilution HDF mode [20]. The volume of the infusion solution in classical HDF is limited and 8–10 L of fluid is most commonly used—hence, on-line HDF mode is preferred. When on-line prepared fluid is used, convection also reduces the dialysis fluid flow and this fluid serves as the source of substitution fluid. The same randomized studies used higher fluid flow than in standard hemodialysis Qd (553 mL/min in ESHOL Study, 500–600 mL/min in FRENCHIE Study). This means an additional consumption of 70 L of water per week, 910 L per month and 10,920 L per year in on-line HDF mode.

Recent studies have shown that for dialysers with features that promote good dialysate distribution, increasing Qd during standard hemodialysis offers only a modest impact on dialyser performance; conversely, the effects of reducing dialysate flow rate below conventional 500 mL/min have not been studied extensively [21]. In trials published between 1984 and 2015 (11 studies), scientists compared the impact of different Qd (500 vs 300 mL/min, 350, 500 and 800 mL/min [22], 500 vs 400 mL/min [19], 400, 500 and 700 mL/min [2, 23]) on diffusion mass transfer of uremic toxins during clinical hemodialysis. In all studies, the mean weight of patients was similar, less than 70 kg. Six studies investigated the influence of flow changes on Kt/V [9, 20]: five studies showed no significant difference between lower vs higher Qd (400 mL/min vs 500 mL/min or 500 mL/min vs 700 mL/min); only one study (from Spain) reported a 4% statistically significant increase of Kt between Qd 400 and 500 mL/min and a 2.9% increase from 500 to 700 mL/min [12, 24, 25]. Two studies focused on clearances for urea and creatinine [24, 25] and showed that small solute clearance is highly dependent on dialysate flow rate but only when the dialysate flow rate is much less than 200 mL/min. Four different trials demonstrated that beta-2 microglobulin (beta2MG) clearance was independent of Qd [21, 26, 27]. In three studies, scientists did not find significant differences in phosphorus removal between dialysate flow rates of 400 and 500 mL/min [18, 20]. In conclusion, most of the studies did not show the impact of Qd changes on uremic toxins clearance and patients’ outcome. Reduction of Qd from 500 to 400 mL/min could save approximately 24 L of water during 4-h standard hemodialysis for one patient, especially in patients with a body mass less than 70 kg. Hence, it might result in saving 72, 312 and 3744 L of water a week, a month and a year, respectively. On a global ecological scale, it would mean conserving 24 billions of liters of water daily. We need more studies in different populations (e.g. in obese patients) and studies into the effectiveness of HDF taking into account different Qd to be sure that water saving during hemodialysis/hemodiafiltration does not affect the quality of the therapy [28, 29]. We currently do not have enough data to assume that Qd reduction is safe and effective for hemodialysis patients. We do not have the same data to recommend this type of guideline changes although we produce a lot of wastewater using more ultrapure water and the problem of water scarcity is particularly important in areas affected by chronic drought.

Finally, the data presented above are only related to dialysate flow. Modifying priming and rinsing strategies should be carefully considered as a possibility of significant savings or waste of valuable resources.

### Electricity

Hemodialysis is a power-hungry medical intervention. The massive amount of power is used by water, the treatment unit and by HD machines. Hot disinfection of the loop as well as hot disinfection of the dialysis machines are the most energy-consuming processes during renal replacement treatment. It is obvious that proper disinfection is needed to avoid the risk of infections, but we should (re)consider which kind of disinfection is effective and rational from an economical point of view. The modern hemodialysis machines are prepared for providing thermochemical disinfection (84–85 °C). An unsolved issue remains the effectiveness of the distribution loop for hot disinfection. We did not find any studies which proved the advantages of this method. The results of the study NCT01138280—Heat Disinfection of HD Water Treatment System in Hemodialysis Patients has not been posted or published [30]. If we take into consideration that heating of 1 L of water by 1 °C consumes 1.16 Wh, we should reconsider the sense of such non-economical processes. On the other hand, perhaps using alternative energy sources such as solar panels, would make it possible to reduce the use of highly polluting energy sources.

### Medical waste

The problem of waste produced during medical interventions exists in all countries with developed healthcare system. Although many countries introduced legal regulations concerning medical waste management, the problem of the rising quantity of medical waste is growing according to the number of medical procedures provided. Dialysis is one of the medical interventions which produce relatively high amounts of waste. We can estimate that during every HD session even more than 2 kg of potentially infective waste is produced. It means that every year HD providers produce more than 1 billion kg waste. It causes serious economical impact—the cost of 1 kg waste in Europe is about two EUR [31]. Proper selection of waste and using lighter disposables can be directly translated into the cost savings. Complete emptying of the dialysis set plays a key role in reducing the weight of the dialysis waste. Unpublished data from the Fresenius 6008 machine observational study showed that the weight of medical waste can be 150 g lighter in case of a dialysis session provided with the Fresenius 6008 machine in contrast to the Fresenius 5008S or the Fresenius 4008S machines. Using modern HD machines that allow emptying of the dialysis set helps to save about 24 kg of medical waste per patient yearly.

## Conclusion

Environment is now an intricate part of our human life. We are responsible to keep it as much as possible in an unchanged shape for the next generations. Even small changes made in our everyday activities could have a big impact on the ecosystem of our planet. The medical services are one important part of daily human activities that massively influence the current waste burden. Renal replacement therapies are one of the most resource-consuming medical technology. To provide a dialysis session we need large amount of water and electricity. Additionally, every HD session produces a significant number of medical wastes. In addition to the massive effect on the environment, resources needed for HD treatment generate serious costs for the healthcare system. The spending for medical waste utilization and its impact on the environment are undisputed. Every year 1 billion kilograms of medical waste produced during dialysis worldwide must be separated, stored, and burnt. Observance of the recommendations below can help to reduce the impact of HD treatment on the environment and will allow to make significant savings.

Next to the nephrologists and their change of thinking, the industry should increase their efforts in the development of more “green” products—more effective water treatments units, less electricity consuming HD machines, light disposables constructed with less harming components (e.g. phthalanes). The future of our planet is in our hands—even small step can cause the huge improvement.

1. Be aware about the impact of your decision on environment.
2. Segregate the waste. Separate the recyclable and medical wastes.
3. Use lightweight dialysers and other disposables.
4. Empty completely the dialysis set—dialyser and bloodlines. Use HD machines equipped with automatic function of emptying the dialysis sets.
5. Carefully consider the dialysis fluid flow and use priming with dialysis fluid instead of saline.
6. Check the energy consumption of your HD machines. If you plan to replace HD machines, choose models consuming less electricity.
7. Use certified, environment-friendly disposables—without phthalanes and other toxic plasticizers.
8. Carefully plan patients’ travel routes—you will save time, money and decrease CO_2_ emission.
9. Use proper function of HD machine to limit water and electricity consumption.
10. Urgent research efforts to study dialysate regeneration, and waste disposal, by new methods should also be considered.
